# Relationsheep between life satisfaction and instagram addiction among Tunisian doctors

**DOI:** 10.1192/j.eurpsy.2023.1410

**Published:** 2023-07-19

**Authors:** S. Kolsi, N. Messedi, I. Chaari, A. Guermazi, F. Charfeddine, L. Aribi, J. Aloulou

**Affiliations:** Psychiatry « B » Department, Hedi Chaker University Hospital Center, Sfax, Tunisia

## Abstract

**Introduction:**

Instagram is currently the fastest growing social networking site in the world. Its image-driven nature might encourage users to mainly share positive and idealized moments of their lives.

**Objectives:**

To study the link between instagram addiction and the life satisfaction among tunisian doctors.

**Methods:**

This was a cross-sectional, descriptive and analytical study conducted on 106 doctors during the months of september and october 2022. We used:

- An anonymous self-questionnaire via google-forms in order to collect data that co-occur the use of instagram.
- Instagram Addiction Scale (IAS) : to assess Instagram addiction levels. A score above 37 indicates addiction to instagram.
- The Satisfaction With Life Scale (SWLS) : to measure an individual’s global satisfaction with life. Higher score displayed higher satisfaction with life.

**Results:**

The study included 106 doctors.

The mean age was 32.32 years (SD=5.66 years) and the sex ratio (M/F) was 0.60.

The mean score for addiction to instagram was 31.18 (SD=11.64). The prevalence of instagram addiction was : 42.5%.

The mean life satisfaction score was 20.43 (± 4.21). More than forty percent (42.5%) were slightly satisfied and 30.2% were slightly dissatisfied.

The instagram addiction was correlated negatively with life satisfaction (r=-0.292; p=0.002).

Instagram addiction was associated witn low satisfaction (p=0.0001).

Among addicted doctors, 53.3% were slightly dissatisfied.

Among non-addicted doctors, 55.7% were slightly satisfied.

**Conclusions:**

Our findings suggest that the higher the intensity of using instagram the lower the life satisfaction.

So, interventions targeting addiction to instagram should be integrated in order to ameliorate life satisfaction and quality of life.

**Disclosure of Interest:**

None Declared

